# How to Prevent Mammary Abscess

**Published:** 1879-12

**Authors:** 


How to Prevent Mammary Abscess.—It is not well to wait until there are marked evidences of inflammation. A good rule is to ask the patient if the breast feels heavy and drag so much to the side as to impede the movements of her arms, and particularly to prevent her from bringing her arms close to the side of the body. This being the case, take two or three broad strips of plaster (which, if good, will not need heating), direct the nurse to lift the breast gently toward the medium line of the body upon the sternum, then apply one strip on the lower half of the breast, the other on the upper half, reaching from one axilla to the other. Other auxiliary strips may be applied according to the size of the breasts and effectiveness of the first two. When the breasts have become "caked," and the patient is so uncomfortable as to be unable to sleep, this procedure will generally be followed by immediate relief from discomfort, and the milk will oftentimes flow from the nipple in streams.—*Med. Herald.*

